# Cincinnati Medical Society

**Published:** 1880-06

**Authors:** 


### CINCINNATI MEDICAL SOCIETY.

*Tuberculosis as an Infectious Disease.*—Dr. Bruhl made some remarks on this subject. He said Professor Cohnheim's investigation had established it beyond a doubt that tuberculosis belongs to this class of disease. From observations in his practice he had suspected this fact years ago and advanced this theory to his friend, Dr. Richardson, late professor in the Miami Medical College, who concurred in his views. Several years ago he had brought the subject before the German Medical Society of this city and asked the members as to their experience at the bedside. Now since experimental pathology had run ahead of clinical injury, it behooved the practicing physician to pay attention to this subject, as it was a matter of great importance and might furnish the means of preventing the disease to some extent. For this reason he considered it proper to bring before the society some cases, partly taken from the practice of others, and partly from his own observations, which seem to verify the results of the pathological experiments. An article bearing on this subject was published some time ago in the *Berliner Klinische Wochenschrift* by Dr. Hubert Reich, the prominent points of which might be summed up as follows:

I. From July 11, 1875, till September 29, 1876, there died in the village of Neuenburg, which is situated on a high bluff of the Rhine and enjoying excellent hygienic conditions, ten children with tubercular meningitis, who had been born in the period between April 4, 1875 and May 10, 1876.

II. A hereditary disposition to this disease did not exist in any one of the children.

III. All these children were delivered by the same midwife.

IV. This midwife was suffering from pulmonary tuberculosis, and a careful examination made in the month of July, 1875, revealed the presence of caverns and sanio-purulent sputa. She died July 23, 1876, having attended to her duties till a short time before her death.

V. She had the bad habit, when a child was born, to remove the phlegm from the respiratory passages by aspiration with her mouth and in slight cases of asphyxia to blow air into the child's mouth in order to more vigorously excite the respiratory motions, whereby the transmission of the air expired by her and perhaps of particles of her sputa to the lungs of the children become probable.

VI. Tubercular meningitis is no disease endemic in the village. In the nine years antedating this event (1866—1874) only two children out of ninety-two died with this disease in their first year, and in 1877 out of twelve only one, and this descending from tuberculous parents.

Taking all these facts into consideration it could not be assumed that all these cases of death from tubercular meningitis, in such a short space of time, were a mere accidental accumulation of cases, as Virchow has observed it, but they pointed rather to a common source and origin, and this source seemed to be the infection emanating from the phthisical midwife.

To these cases the speaker added his own observations. More than a dozen times he had observed that after a man had died with tuberculosis, his wife, marrying again, a few years later had died with the same disease, and the same had been the case with her second husband. Or a wife had died, her husband had married again, and then he, and later, the second wife had succumed with phthisis. In all these cases there was, as must be recollected, no hereditary disposition in the second set of victims. A more remarkable case still the speaker had at present under his treatment. It was that of a gentleman, twenty-four years old, who was last year a pupil of a literary college, healthily located. Two of his intimate friends, inmates of the same institute, had suffered and died with tuberculosis and he, much attached to them, had assisted in nursing them. Shortly after he was attacked by a severe obstinate cough, which resisted all treatment. When vacation commenced he went into the country and there he finally improved. Last winter his cough had set in violently again, and he presented at present all the symptoms and signs of tuberculosis. His parents were hearty and healthy, so were his sisters and brothers. There was another case deserving attention. The speaker knew a family, where both parents enjoy exceptionally good health, being over seventy years of age, four of their children out of five had died with pulmonary tuberculosis. How could this fact be accounted for? If by infection, how was the first of the victims infected? Perhaps in a similar manner as the young gentleman now under the the speaker's treatment. But why were neither of the parents infected? Because by their robust health their power of resistance was greater. There was, he said, a parallel in syphilis. Not everyone exposing himself to the syphilitic virus became infected.

The speaker presented still another fact for consideration. He had been physician in several religious institutions, and had been struck by the greater number of deaths from pulmonary tuberculosis occurring amongst the inmates. Could it be attributed to close confinement, bad ventilation, overworking, poor nourishment? Not at all. Flour gardens were attached to all of these institutions where the inmates could enjoy fresh air, their rooms were well ventilated and kept extremely clean. Their diet was nourishing and plentiful, their duties not too heavy. The novices would enter apparently healthy and from well-to-do parents. He could not comprehend why the inmates should be more subject to the disease than those outside, if it were not attributable to infection occasioned by the inmates afflicted with it.

In connection with these facts the speaker proposed the question to those members of the society who practiced in the hospitals; what was their experience as to the nurses of the phthisical patients?

He concluded by saying that he made three remarks rather for obtaining than for giving information and that they were necessarily incomplete.

Dr. Rothaker stated, in answer to the question as to whether any tubercular infection had ever been noticed among the nurses at the Cincinnati Hospitals, that he had known some of them for about eight years, and they had shown no signs of infection. The rooms where the nurses had slept were so arranged that the same air breathed by the patients was also inhaled by them; and it was stated that the male and female wards had each not less than twenty consumptive patients all the time, though other patients representing various diseases were treated in the same wards. Mr. Hodge, one of the male nurses, had been in the house about eight years performing all the duties required of the nurse and yet had never presented the slightest symptom of tuberculosis. The same could be said of a nurse for the female department. She had been acting in that capacity for ten years and yet was as robust as ever. As to the infectious nature of tuberculosis the speaker himself was not decided, but thought that if such a view was to be expected by the profession it would cause a great revolution in the management of such cases.

The same speaker reported the result of several post mortem examinations made within the last few days at the Cincinnati Hospital, which, by the kindness of Dr. Hurt, we are enabled to print in full.

*Echinococci of the Liver with Cancer of the Pylorus.*—Henry Spelker; fourteen hours after death. *Post mortem* rigidity slightly marked; heart small; weight 7 ounces.

Scattered throughout the lungs were a number of small nodules, from the size of a pea to that of a walnut; the bronchial glands were enlarged and caseous. There was half a pint of fluid in the left pleural cavity, and one pint in the right.

The entire pyloric portion of the stomach, together with the surrounding tissues, was included in a dense new growth having all the gross characters of cancer. The little finger could, with difficulty, be pushed through the pyloric orifice. In the right lobe of liver, anteriorly, near gall bladder was found a growth about the size of a hen's egg embedded in the liver and projecting slightly above the surrounding tissue. On section, this growth presented a number of alveoli and cysts of various sizes, having all the characteristic appearances of a multilocular cyst of echinococci. Microscopical examination afterwards confirmed this by the appearance in the fluid of hooklets, etc.

The mesenteric glands were enlarged and very hard, and on section presented the same appearance as the section of the pyloric extremity of the stomach. Other organs normal.

*Intestinal Obstruction.*—Patrick McLaughlin; autopsy sixteen hours after death. Subject large and muscular, rigor mortis well marked. Omentum filling left inguinal canal and firmly attached there.

Right inguinal canal contained an old hernial sac, but neither bowel nor omentum.

Bowels deeply conjested, some lymph and purulent fluid in peritoneal cavity. It was discovered that the vermiform appendix had become attached to the right lateral abdominal wall, and through the loop thus formed, several coils of the ileum had passed and had become firmly strangulated.

The loop lay immediately in line with the internal abdominal ring, and it is probable that when the reduction of the hernia was affected (which was accomplished by the patient himself) the bowel was pushed along the muscular structures of right iliac fossa, entering the loop.

Friends would permit no further examination.

*Intestinal Obstruction.*—Lina Beckman; autopsy seventeen hours after death. *Post mortem* rigidity exceedingly slight. An opening the size of a three cent piece at the junction of Poupart's ligament and anterior superior spinous process of ileum of left side from which oozed a small quantity of pus. A bougie was inserted, which passed its entire length into a fistulous tract.

Lungs normal with exception of hypostatic conjestion. Heart small, otherwise normal. Intestines inflated with gas, containing a coffee-colored fluid. Greater omentum adherent by inflammatory bands, at a point immediately above and to the left of promontory of sacrum; from this point another band stretched across the junction of rectum with signoid flexure and adherent in left iliac fossa.

The coil of intestine immediately above the part girded by the omentum was of a purple color and the veins everywhere over full. This coil included part of first and entire second foot of ileum. The intestine on the ileo-cæcal side of stricture was empty and collapsed. The top of index finger could with difficulty be passed under the girding band.

Large intestine, when abdominal cavity was first opened, was exceedingly small in size, appearing to be about one-third the size of small intestines.

On examining the parts of attachment of the omentum, the peritoneum was found roughened, discolored and covered with organized lymph. The mucous membrane of the strictured coil of intestine was very dark in color, exhibiting marked venous stasis and œdema. The neighboring coils showed active congestion. The peritoneum over the posterior abdominal wall had to be divided before the bougie mentioned above could be discovered. It was found that the psoas muscle was hollowed out into a suppurating cavity filled with the debris of a broken down tissue; the cavity extending in an upward direction along the side of the vertebral column to about the middle dorsal region; the tract of the abscess was bounded and limited by a dense wall of connective tissue, almost cartilaginous in consistence; this tissue wrapping in the front and lower portion of left kidney, involving the connective tissue about the left kidney. The abscess had in no direction a connection with bone, and appeared to have originated spontaneously in the psoas muscle and not in the pelvic cellular tissue.

The left kidney was so pressed upon that its tissue was extensively destroyed; the bases of the pyramids at some points almost touching the capsule; weighed 4½ ounces; the right kidney weighed 17½ ounces, and presented on section a somewhat cunjested appearance of the pyramids and with a fatty color in the cortex; there was no distinct reaction with iodine. The spleen exhibited sago joints, but no reaction with iodine. Brain not examined.

*Fracture of Spine, etc.*—Peter Wagner; injured by locomotive eight days before death. *Post mortem* rigidity slight. An incision was made over the spinous processes from the seventh cervical vertebra to the sacrum. The subcutaneous muscles and tissues were infiltrated with blood; the spine below the tenth dorsal vertebra was displaced to the right; the lateral parts of the eleventh dorsal, together with the heads of the twelfth rib of each side were comminuted.

This fracture, viewed from the front, was seen to pass through the body of the eleventh dorsal, so that the spine was divided into two parts; each could be moved upon the other. The fifth rib of right side was broken through its cartilage near the junction with rib. The seventh rib of right side was broken near its angle, as was also the fifth rib of left side. The seventh, eighth and ninth left ribs were partially broken (green stick fracture) at a distance of about 6 or 7 inches from their anterior extremities; the seventh and eighth left ribs were broken through the cartilages and displaced upon the ribs immediately above. No fragments penetrated the pleuræ or lungs. There was an extensive sub-pleural extravasation of blood on right side, involving a thin stratum of lung tissue. On the left side the central portions of lung tissue were consoldated and showed traces of extravasation of blood. Liver somewhat fatty. Left kidney showed thickening of the lining of the pelvis, with a thin layer of false membrane upon it, with some pus in the calices. The cortex exhibited points of fatty degeneration. Right kidney normal. Bladder considerably distended. Other organs normal. Brain not examined.

On removing the spine the cord was found completely divided, the upper and lower fragments being connected by a small portion of dura mata which remained uprup-tured.

*Syphilitic Ulceration of the Larynx.*—Herman Gold; autopsy thirty-six hours after death. *Post mortem* rigidly well-marked. Ulceration in larynx had penetrated the mucous membrane and inclosed the right arytenoid cartilage in such a manner that it lay loosely within the cavity, and could be picked out without difficulty. A point of ulceration, not so deep as this, was in the opposite side, immediately above the true vocal cord. There was evidence, from the appearance of the epiglottidean fold, of œdema during life. Membrane lining the larynx was thickened and covered with a tenacious mucus. Condition of blood in heart, lungs, and kidney indicated excess of carbonic acid; color of blood being dark, and *post mortem* staining extensive.

